# Dynamics of Soil Bacterial Communities in Response to Repeated Application of Manure Containing Sulfadiazine

**DOI:** 10.1371/journal.pone.0092958

**Published:** 2014-03-26

**Authors:** Guo-Chun Ding, Viviane Radl, Brigitte Schloter-Hai, Sven Jechalke, Holger Heuer, Kornelia Smalla, Michael Schloter

**Affiliations:** 1 Julius Kühn-Institut - Federal Research Centre for Cultivated Plants (JKI), Institute for Epidemiology and Pathogen Diagnostics, Braunschweig, Germany; 2 Helmholtz Zentrum München, German Research Center for Environmental Health, Research Unit for Environmental Genomics, Neuherberg, Germany; Graz University of Technology (TU Graz), Austria

## Abstract

Large amounts of manure have been applied to arable soils as fertilizer worldwide. Manure is often contaminated with veterinary antibiotics which enter the soil together with antibiotic resistant bacteria. However, little information is available regarding the main responders of bacterial communities in soil affected by repeated inputs of antibiotics via manure. In this study, a microcosm experiment was performed with two concentrations of the antibiotic sulfadiazine (SDZ) which were applied together with manure at three different time points over a period of 133 days. Samples were taken 3 and 60 days after each manure application. The effects of SDZ on soil bacterial communities were explored by barcoded pyrosequencing of 16S rRNA gene fragments amplified from total community DNA. Samples with high concentration of SDZ were analyzed on day 193 only. Repeated inputs of SDZ, especially at a high concentration, caused pronounced changes in bacterial community compositions. By comparison with the initial soil, we could observe an increase of the disturbance and a decrease of the stability of soil bacterial communities as a result of SDZ manure application compared to the manure treatment without SDZ. The number of taxa significantly affected by the presence of SDZ increased with the times of manure application and was highest during the treatment with high SDZ-concentration. Numerous taxa, known to harbor also human pathogens, such as *Devosia*, *Shinella*, *Stenotrophomonas*, *Clostridium*, *Peptostreptococcus*, *Leifsonia*, *Gemmatimonas*, were enriched in the soil when SDZ was present while the abundance of bacteria which typically contribute to high soil quality belonging to the genera *Pseudomonas* and *Lysobacter*, *Hydrogenophaga*, and *Adhaeribacter* decreased in response to the repeated application of manure and SDZ.

## Introduction

The use of animal manure for fertilization of agricultural soils has a long tradition in many parts of the world and is generally assumed to be ecologically more friendly and sustainable than mineral fertilizer. In particular organic and bio-dynamic farms depend on manure or compost as sources for fertilization. However, industrial husbandries with typically large numbers of animals sharing limited space depend on the prophylactic and therapeutic use of antibiotics. In addition, in several parts of the world antibiotics are still being used as growth promoters. Although antibiotics as growth promoters were banned in many European countries, considerable amounts of antibiotics such as tetracyclines, β-lactams and sulfonamides are still used in animal husbandries [1]. Depending on their physicochemical properties, many antibiotics such as sulfonamides are to a large extent excreted via urine or feces and are not or only to a low extent degraded during manure storage [2], [3]. Thus, spreading manure on agricultural soils does not only introduce nutrients required for maintaining the soil fertility but also antibiotics, their metabolites and antibiotic resistant bacteria. Indeed, antibiotics have been detected in the environment due to the use of manure for soil fertilization or direct deposition via dung and urine of animals grazing on pastures [4], [5].

The rapid sequestration of most antibiotics in soils, as e.g. observed for the sulfonamide antibiotic SDZ [6], leads usually to low concentrations of bioavailable SDZ, which are far below the minimal inhibitory concentrations after a single application of manure. Most data published indicated only short-term effects on the microbial community after a single application of manure spiked with antibiotics followed by a fast regeneration of the community structure [7], [8] and its function [9]–[11]. However, agricultural management implies in most cases a repeated application of manure mainly during the vegetation period to keep the level of nutrients needed for best possible plant growth. Surprisingly, little is known of the effects of repeated application of manure containing antibiotics on microbial communities in soil which might cause cumulative effects. Despite a rapid dissipation and sequestration of SDZ in soil [3], [6], [12] an increased abundance of *sul1* and *sul2* resistance genes and their transferability was observed in soils treated with manure containing SDZ compared to control manure under microcosm, mesocosm and field conditions [13]–[15], suggesting that manure containing antibiotics enhanced the spreading of the antibiotic resistance genes in soils. In particular the repeated application of manure in combination with antibiotics may set the ground for an increased abundance of resistant bacteria as recently reported by Heuer *et al.* [16] and thus might stimulate the spreading of antibiotic resistance genes and mobile genetic elements in agricultural ecosystems. Antibiotic resistance genes localized on mobile genetic elements can be captured by human and veterinary pathogens and thus pose a threat to the treatment of bacterial diseases [17]–[19].

In the present microcosm experiment, the effect of repeated application of manure containing antibiotics on the soil bacterial community was investigated by 454 sequencing of 16S rRNA gene fragments amplified from total community (TC)-DNA. We have chosen the sulfonamide SDZ as a model compound as it is still frequently used in pig husbandry [4] and highly persistent in manure [20]. Manure unspiked and spiked with SDZ in two concentrations was applied three times, and the bacterial community composition was monitored over a period of 193 days. We hypothesized that repeated application of manure spiked with SDZ to soil would increase the degree of disturbance and reduce the resilience of soil bacterial communities compared to a single application of manure spiked with SDZ or soils treated with manure not containing antibiotics. Resilience was measured as the stability of bacterial diversity after changes in soil properties ([21]; here manure addition). To differentiate between short and long-term effects, the soil was sampled 3 and 60 days after each manure application, respectively.

## Materials and Methods

### Experimental design

The experimental design is described in detail by Heuer *et al.* [16]. In short, topsoil samples (Ap horizon) of a silt loam soil (Orthic Luvisol) with no history of manure application were used. Pots were filled with 100 g of 2 mm-sieved soils and treated with 4 g of manure from healthy pigs (approximately 30 m^3^/ha) spiked with SDZ or not to achieve a SDZ concentration in soil of 10 mg/kg, 100 mg/kg, or no SDZ (Sigma-Aldrich, Germany), which corresponded to samples S10, S100, and S0, respectively. Soil samples without manure application served as controls (U). To avoid effects based on different manure composition at the different time points of application, manure was taken before the experiment and frozen in aliquots at −20°C. For each time point of application, an aliquot of the manure was thawn and the corresponding amount of SDZ was directly applied before mixing the manure into the soil. The SDZ concentration S10 was chosen in accordance to maximal inputs of sulfonamides detected in agricultural soils [3], [22]. Manure loads were applied on days 0, 63 and 133. The loosely covered pots were incubated at 15°C in the dark. Water was added to the microcosms twice a week to compensate for weight losses and to maintain a soil moisture of about 55% of the soil's water-holding capacity. For each treatment and time point 5 replicates were prepared and treated individually for the subsequent analysis. Samples were taken every 3rd and 60th day after manure application (days 3, 60, 66, 123, 136 and 193) and kept at −80°C until further analysis. No specific permissions were required for collecting the manure and soil and the study did not involve endangered or protected species.

### DNA extraction

Extraction of total community DNA from soil samples was performed according to Griffith *et al.* [23]. Briefly, 0.4 g soil per replicate were added to a lysing matrix tube (MP Biomedicals, Germany) and submitted to phenol∶chloroform extraction starting with homogenization for 30 seconds at 5.5 m S-1 (Precellys, PeqLab, Germany). Extracted DNA was finally resolved in 50 µl DNase-free water. Quality and quantity of DNA extracts were checked using a spectrophotometer (Nanodrop, PeqLab, Germany). For the extraction of total community DNA from manure, 10 ml manure were centrifuged at about 6500 *g* for 10 min, the pellet was homogenized and DNA was extracted from 0.5 g manure pellet using the FastDNA SPIN kit for soil (MP Biomedicals, Heidelberg, Germany), followed by a purification step using the Geneclean spin kit (MP Biomedicals, Heidelberg, Germany), according to the manufacturer's instructions.

### Preparation of the 16S rRNA gene amplicons, pyrosequencing and data processing

To generate the amplicon library for *Bacteria*, 16S primers 926-F (5′-AAACTYAAAKGAATTGACGG-3′), *Escherichia coli* position 907–926 and 630-R (5′-CAKAAAGGAGGTGATCC-3′), *E. coli* position 1528–1544 [24], were selected. Fusion primers were designed according to the guidelines of ROCHE (www.my454.com), by extending the specific primers with a 10 base multiplex identifier (MID), a 4 base key and the respective sequencing primers A or B for bidirectional sequencing. The optimal PCR conditions were determined by gradient PCRs with annealing temperatures ranging from 50°C to 60°C. Amplicons were finally generated using 50 ng of extracted DNA, an annealing temperature of 50°C and 22 PCR cycles. The PCR products were purified with AMPure Beads (Agencourt, Beckman Coulter, Krefeld, Germany) according to the Amplicon Library Preparation Method Manual (www.my454.com) and pooled in equimolar amounts.

Sequencing of the 16S rRNA genes was performed on a second-generation pyrosequencer (454 GS FLX Titanium, ROCHE, Germany) following the manufacturer's protocol for amplicon sequencing (www.my454.com). The number of replicates per treatment included in the final sequencing run can be found in Table S1 in File S1.

The automatic amplicon pipeline of the GS Run Processor (ROCHE) was used to perform an initial quality filtering of the pyrosequencing raw reads in order to remove failed reads, low quality reads and adaptor sequences. Sequence files were submitted to the NCBI Sequence Read Archive (www.ncbi.nlm.nih.gov/sra/) and are available with the study accession number SRP038712.

### Sequence analysis

The two data sets acquired by the forward and reverse primers were analyzed separately to avoid the systematical bias of individual primers. The analyses were mainly performed according to Ding *et al.* [25]. Only those sequences with a length above 200 bp after removing the barcode, primer and unpaired regions were subjected to further analysis. The unpaired regions were truncated based on a standalone BlastN analysis against a bacterial database described by Pruesse *et al.* [26]. Sequences were grouped into operational taxonomic units (OTUs>97% sequence identity) using software package mothur (v1.14.0). The classification of sequences was performed using software RDP MultiClassifier at >80% confidence [27]. A taxonomic OTU report with each row representing one OTU containing taxonomic positions (from phylum to genus) and the number of sequences for each sample was constructed based on the OTU assignment and on the classification.

Analyses based on the taxonomic OTU report were done with R (version 2.14). To allow the comparison of samples with different number of reads, all algorithms selected rely on relative abundance of taxonomic groups. To check whether the forward and reverse primers revealed a similar taxonomic composition or not, the percent of Bray-Curtis similarity of taxonomic composition between two data sets was calculated for each sample based on the percent of classified taxa at phylum, class, order, family, and genus level using the R add-on package “vegan”. To evaluate the influence of repeated application of manure containing SDZ on the bacterial community compositions, the pairwise Pearson dissimilarities between S0 and S10 or between S0 and S100 for day 193 were calculated. To compare the microbial community composition between samples, non-metric multidimensional scaling (NMDS) analysis was carried out based on the Pearson dissimilarity matrix using R package MASS. The 3-dimensional plots of NMDS analysis were created with the R package “rgl” in conjunction with the Linux command “import”. Tukey's honest significance tests under a generalized linear model via a logistic function for binomial data with the package multcomp [28] was applied to identify the discriminative taxa between treatments using a Bonferroni adjusted *p* value<0.05.

## Results

### Effects of the repeated application of manure containing SDZ on the composition of soil bacterial communities

In this study, bacterial 16S rRNA gene fragments amplified from TC-DNA extracted from 50 composite soil samples were sequenced using forward (926F) and reverse (630R) primers. A total of 132,278 sequences for 50 samples with 790–5685 high quality sequences per sample were obtained with the forward primer, and 162,446 sequences for 49 samples with 1105–7053 high quality sequences per sample with the reverse primer.

**Figure 1:**
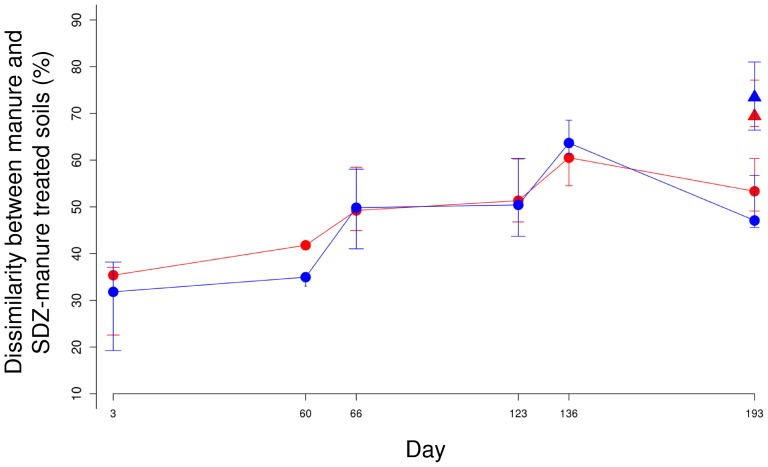
Dissimilarity between soils treated with manure (S0) and manure spiked with SDZ (S10 indicated by circles; S100 indicated by triangles) at different sampling times. Red symbols: results based on the data set acquired by the forward primer; blue symbols: results based on the data set acquired by the reverse primer. Error bars indicate the first and third quartiles.

**Figure 2:**
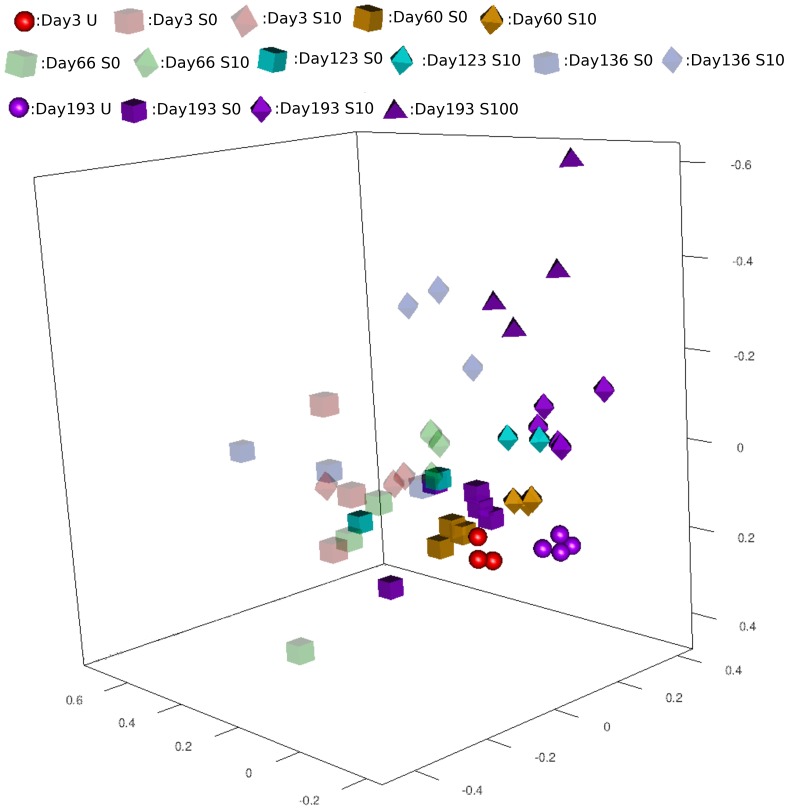
Non metric multidimensional scaling based on OTU reports acquired by the reverse primer sequencing. Balls: untreated soils; cubes: S0; octahedron: S10; pyramid: S100.

To study the effect of repeated applications of manure containing SDZ on the soil bacterial community composition, 16S rRNA gene pyrosequencing data sets acquired from TC-DNA of soils treated with SDZ manure or control manure were compared. Sequences obtained with the forward and reverse primers were treated independently and grouped into OTUs (>97% sequence identity). Both datasets revealed similar changes of bacterial community composition after manure application (Figure 1). In general, the Pearson dissimilarities of the bacterial community compositions between the S0 and S10 soils increased with repeated manure application (Figure 1). Shortly after each manure application the dissimilarity between the bacterial community composition of S0 and S10 soils was strongly increased. Sixty days after the first and second manure applications the dissimilarity remained at the same level as shortly after manure application, while 60 days after the third application a decrease in dissimilarity between S0 and S10 soil was observed. Compared with the S10 soils on day 193, the S100 soils were less similar to S0 soils in the bacterial community composition, indicating that a higher concentration of SDZ caused more pronounced changes of the soil bacterial communities (Figure 1). Non-metric multidimensional scaling based on the OTU reports acquired for the forward (Figure S1 in File S1) and reverse (Figure 2) data set confirmed the findings presented in Figure 1 as it showed that the bacterial community composition of S10 soils increasingly deviated from the S0 soils with time, and this difference was even more pronounced for S100 soils. Furthermore, the faint color symbols in Figure 2 representing the bacterial community composition shortly after manure application strongly deviated from the community composition of untreated soil suggesting a pronounced disturbance of the system. In contrast to S10 and S100 soils, the bacterial community composition of S0 soils (indicated by cubes) 60 days after the third manure application still grouped in the vicinity of the untreated soils (Figures 2 and S1) suggesting a stronger resilience of the soil bacterial community towards manure when no antibiotic was present.

**Figure 3:**
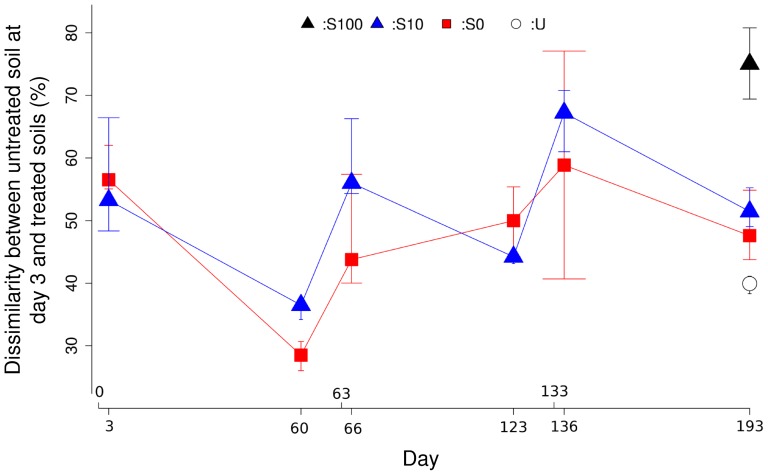
Dissimilarity between soils treated with manure (S0, S10 and S100) or not (U) and the untreated initial soils (Day3 U) at different sampling times. Dissimilarity was calculated from the data acquired by the reverse primer. Error bars indicate the first and third quartiles.

The difference between bacterial communities of the initial soil (day 3 untreated) and of the treated soil was calculated based on Pearson dissimilarity (Figure 3). A pronounced increase in difference was observed for soils shortly after each manure application (S0, S10). The dissimilarity increased with the repeated applications of manure containing SDZ for samples collected 60 days after each manure application. The highest deviation was observed for soil treated three times with manure containing the high concentration (100 mg/kg) of SDZ. At the same sampling time, the least difference was found for the untreated control soils.

### Taxa with significantly altered relative abundance in response to repeated application of manure spiked with SDZ or unspiked

**Figure 4:**
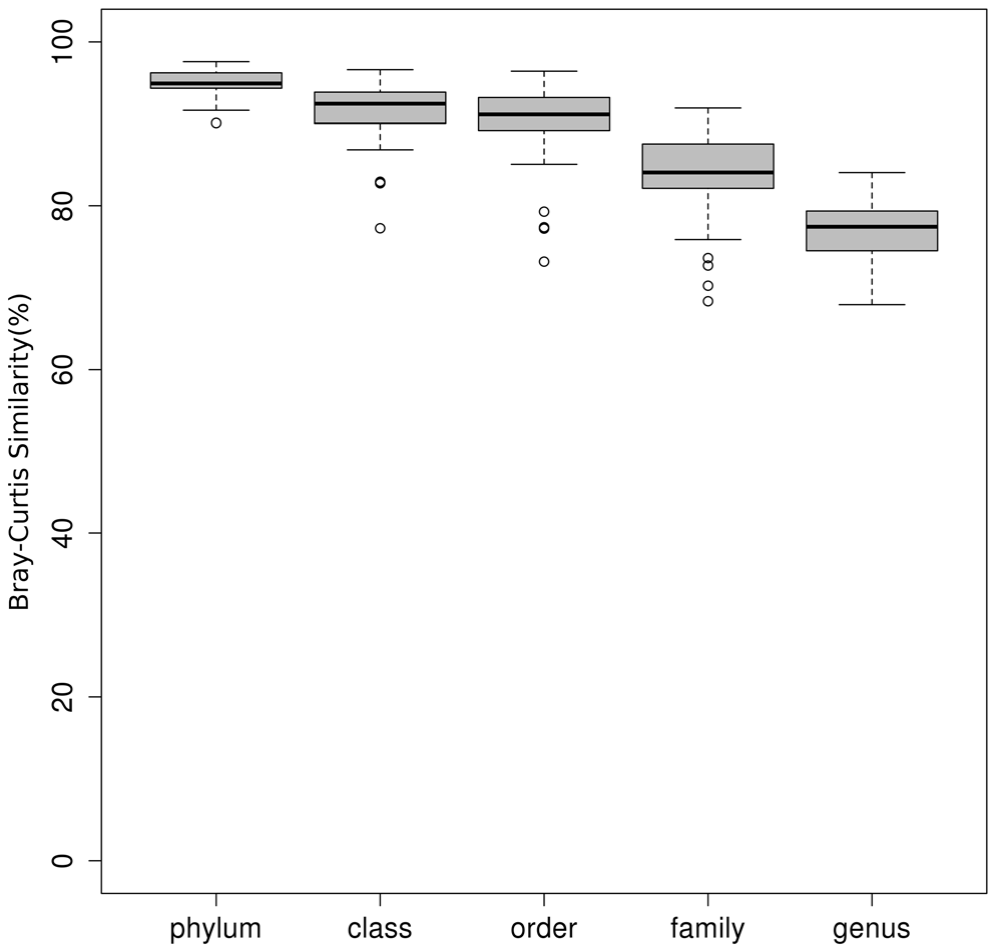
Box plot showing the similarity of taxonomic composition at different ranks between data acquired by the forward and reverse primers.

About 86% of the sequences obtained with the forward primer could be assigned to 21 phyla while 90% of the reverse primer derived sequences were affiliated to 19 phyla. The percent Bray-Curtis similarities of the taxonomic composition between the data sets acquired with the forward and reverse primers, respectively, were calculated for each sample based on the percent of the classified taxa at different taxonomic ranks (from phylum to genus level). Although from the phylum to the genus level the similarity of the taxonomic composition decreased as shown in Figure 4, the overall taxonomic composition of both data sets was very similar.

To identify the taxa which significantly (Bonferroni adjusted *p* value<0.05) responded to the repeated application of manure, comparisons were made between soils treated with S0 or not (U). All taxa with significantly different relative abundance (Bonferroni adjusted *p* value<0.05) were considered. In soil samples taken three days after the addition of manure short-term responders were identified (summarized in Table S2 for the reverse primer derived sequence set and in Table S3 in File S1 for the sequence set obtained with the forward primer). Based on these data (Table S2 and S3) we could mainly identify genera belonging to the *Proteobacteria* such as *Delftia* (*Betaproteobacteria*), *Pseudomonas*, *Pseudoxanthomonas*, *Stenotrophomonas* (*Gammaproteobacteria*), *Bacillus*, *Lactobacillus*, *Streptocococcus*, *Clostridiales* (*Firmicutes*) and *Arthrobacter* (*Actinobacteria*) which were significantly increased in relative abundance in S0 soils compared to the untreated soil as short time responders to the manure application. The increase in the relative abundance of *Firmicutes* (Table S4 in File S1) is likely related to the high abundance of these organisms in the manure (data not shown). In turn the relative abundance of different genera of the *Acidobacteria* and *Planctomycetaceae* but also *Gemmatimonadetes*, the genus *Methylibium* (*Betaproteobacteria*) decreased in S0 soils compared to the untreated soils (Table S2 and S3 in File S1). An increased relative abundance of the genera *Devosia*, *Clostridium*, *Peptostreptococcus* and *Adheribacter* in response to the manure application was observed by comparing U and S0 soil on day 193 (Table S5 and S6 in File S1). In contrast, *Myxococcales* seemed to be negatively affected by manure. On day 193 the relative abundance of *Acidobacteria* was still significantly decreased in all manure treatments compared to untreated soil with the exception of GP10 which showed a higher relative abundance in S10. Some taxa, such as the family *Planctomycetaceae*, were not affected by manure application.

With repeated application of manure the number of taxa with significantly altered relative abundance in S10 compared to S0 soil increased (summarized in Table 1 for the reverse primer and in Table S7 in File S1 for the forward primer). An increased abundance in S10 soils was observed in particular after repeated manure application for the genera *Devosia*, *Sphingobium*, Gp10 and *Gemmatimonas*. Shortly after manure application a significantly increased abundance was observed in S10 compared to the S0 soils for the phylum *Chloroflexi*, the class of the *Deltaproteobacteria* and the orders of *Clostridales* and *Actinomycetales*. Acidobacterial genera Gp4, Gp6 and Gp10 with one exception showed always higher relative abundances in S10 but only in some cases these differences were found to be significant (Table 1). The relative abundances of *Pseudomonas*, *Lysobacter* and *Adheribacter* were lower in all S10 compared to S0 soils indicating that the presence of SDZ exerted a negative effect on these taxa.

**Table 1:** Taxa with significantly different relative abundance between manured soils with or without SDZ treatments shortly after manure addition as revealed by barcoded pyrosequencing using the reverse primer (Bonferroni adjusted p value<0.05).

Phylum	Class	Order	Family	Genus	Day 3		Day 66		Day136	
					S0	S10	S0	S10	S0	S10
*Proteobacteria*	*Alphaproteobacteria*	*Rhizobiales*	*Hyphomicrobiaceae*	*Devosia*	0.2±0	0.3±0	0.5±0	**1.5±0**	1.1±0	**2.4±0**
		*Sphingomonadales*	*Sphingomonadaceae*	*Sphingobium*	0.1±0	0.1±0	0.1±0	0.3±0	0.1±0	**0.4±0**
	*Betaproteobacteria*	*Burkholderiales*	*Comamonadaceae*		**3.8±3**	1.6±0	3.3±1	2.1±0	2.6±1	3±1
			*Oxalobacteraceae*	*Herminiimonas*	0.1±0	0.1±0	0.5±0	0±0	**3±4**	0±0
	*Deltaproteobacteria*				1.1±0	1.2±0	1.4±0	1.3±0	3.1±1	**4.4±2**
	*Gammaproteobacteria*	*Pseudomonadales*	*Pseudomonadaceae*	*Pseudomonas*	**0.8±1**	0.2±0	**2.6±4**	0.2±0	0.6±1	0±0
		*Xanthomonadales*	*Xanthomonadaceae*	*Lysobacter*	**2.1±1**	1.2±0	**4.1±1**	0.8±1	**1.7±1**	0.2±0
				*Stenotrophomonas*	**1.6±2**	0.1±0	0±0	0±0	0.1±0	0.3±0
*Acidobacteria*	*Acidobacteria_Gp10*			*Gp10*	1±0	1.5±0	1.7±0	**3±1**	1±0	**2.1±0**
	*Acidobacteria_Gp4*			*Gp4*	3.3±0	**4.7±1**	4.8±1	5.3±0	4.2±2	4.1±2
	*Acidobacteria_Gp6*			*Gp6*	8.7±1	10.5±2	12.1±2	**16.1±1**	8.7±4	9.4±2
*Firmicutes*	*Bacilli*	*Lactobacillales*			5.4±1	5.3±2	3.9±1	**6.3±2**	2.8±1	3.8±1
	*Clostridia*	*Clostridiales*			5.9±2	6.4±1	6±1	8.2±1	7.5±1	**10.5±1**
*Actinobacteria*	*Actinobacteria*	*Actinomycetales*			7.1±1	5.8±2	2.9±1	3.3±1	4.7±5	**5.9±6**
*Bacteroidetes*	*Sphingobacteria*	*Sphingobacteriales*	*Cytophagaceae*	*Adhaeribacter*	**1.5±0**	0.7±0	**2.3±1**	0.6±0	**1.5±0**	0.4±0
*Chloroflexi*					3.3±1	3.6±0	1.9±1	3.8±1	4.5±0	**6.2±1**
*Gemmatimonadetes*	*Gemmatimonadetes*	*Gemmatimonadales*	*Gemmatimonadaceae*	*Gemmatimonas*	0.2±0	0.3±0	0.3±0	0.5±0	0.4±0	**1±1**

Bold numbers indicate taxa with significantly higher relative abundance in SDZ treated or non treated soils. Soils were collected three days after each treatment.

Samples taken 60 days after the first, second and third manure applications mainly revealed the same taxa with increased or decreased relative abundance in S10 compared to S0 as in the samples taken shortly after manure application (shown in Table 2 for the reverse primer and in Table S8 in File S1 for the forward primer). With repeated application of manure (S0, S10) the number of taxa with significantly different relative abundance increased from 3 (60 days after the first manure application) to 11 (60 days after the third manure application). Although not significant at all time points the genera *Devosia*, *Gemmatimonas* and *Acidobacteria* Gp6 and Gp10 showed an increased relative abundance in S10 compared to S0 soils. In S100 soils analyzed only for the samples taken 60 days after the third manure application (day 193) the relative abundances of *Devosia* and *Gemmatimonas* were increased compared to their abundance in S10 and S0 soils clearly indicating that bacteria belonging to these genera take profit from high concentrations of SDZ. A higher relative abundance in S100 soils was also observed for *Anerolineaceae* (*Chloroflexi*), *Microbacteriaceae* and the genus *Clostridium*. Most striking was the selection of *Stenotrophomonas* in S100 soils as sequences of this genus were not detected in S0 and in very low relative abundance in S10 soils on day 193. Also acidobacterial genera were identified as responders to SDZ. However, the different genera of *Acidobacteria* responded differently to SDZ manure. The S10 soils had a higher abundance of *Acidobacteria* belonging to the genera Gp10 and Gp6 (except day 60) compared to S0. On day 193, the relative abundance of Gp4, Gp6 and Gp10 was lower in S100 than in S10 soil. The negative effect of SDZ on *Pseudomonas*, *Lysobacter* and *Adheribacter* was also detected in S100 but there was no significant difference in their relative abundance between S10 and S100.

**Table 2:** Taxa with significantly different relative abundance between manured soils with or without SDZ treatments as revealed by barcoded pyrosequencing using the reverse primer (Bonferroni adjusted p value<0.05).

Phylum	Class	Order	Family	Genus	Day 60		Day 123		Day 193		
					S0	S10	S0	S10	S0	S10	S100
*Proteobacteria*	*Alphaproteobacteria*	*Rhizobiales*	*Hyphomicrobiaceae*	*Devosia*	0.4±0	**1.5±0**	1.8±1	2.2±0	1.5±0b	2.6±1a	3.5±1a
			*Rhizobiaceae*		0.4±0	0.3±0	0.3±0	0.5±0	0.6±0a	0.4±0ab	0.4±0b
		*Rhodospirillales*	*Rhodospirillaceae*	*Magnetospirillum*	0.4±0	0.4±0	0.3±0	0.6±0	0.8±0ab	0.9±0a	0.5±0b
		*Sphingomonadales*	*Sphingomonadaceae*		4±1	3.4±1	4±1	3.4±1	5.4±1a	3.6±0b	3.4±1b
	*Betaproteobacteria*	*Burkholderiales*	*Alcaligenaceae*		0.1±0	0.1±0	0.1±0	0.1±0	0.1±0b	0.1±0b	0.8±0a
			*Burkholderiales_incertae_sedis*	*Methylibium*	1.8±0	1.3±0	1.2±0	1.3±0	2±1a	1.8±1a	0.9±0b
			*Comamonadaceae*	*Hydrogenophaga*	0.1±0	0.1±0	0.6±0	0.2±0	0.5±0a	0.1±0b	0±0b
			*Oxalobacteraceae*	*Herminiimonas*	0±0	0±0	**1.2±0**	0±0	0.5±0a	0±0ab	0±0b
		*Methylophilales*	*Methylophilaceae*		0.1±0	0.1±0	0.3±0	0.1±0	0.3±0a	0.1±0ab	0±0b
		*Rhodocyclales*	*Rhodocyclaceae*	*Shinella*	0±0	0±0	0±0	0.1±0	0±0b	0.2±0b	1.2±1a
	*Gammaproteobacteria*	*Pseudomonadales*	*Pseudomonadaceae*	*Pseudomonas*	0.5±1	0.1±0	0.1±0	0±0	1.2±2a	0.2±0b	0±0b
		*Xanthomonadales*	*Xanthomonadaceae*	*Lysobacter*	**1.6±0**	0.7±0	**1.7±1**	0.6±0	0.9±0a	0.2±0b	0.1±0b
				*Pseudoxanthomonas*	0.7±1	0.4±0	**1.9±1**	0.4±0	0.7±0b	0.4±0b	2±2a
				*Rhodanobacter*	0±0	0±0	0.3±0	0.3±0	0.3±0b	0.9±1a	0.2±0b
				*Stenotrophomonas*	0±0	0±0	0.1±0	0.3±0	0±0b	0.2±0b	3.5±3a
	*Deltaproteobacteria*	*Myxococcales*	*Haliangiaceae*	*Haliangium*	0.5±0	0.1±0	**0.8±0**	0.1±0	0.8±0a	0.1±0b	0.1±0b
			*Nannocystaceae*		0.3±0	0±0	0.2±0	0±0	0.3±0a	0.2±0ab	0±0b
			*Polyangiaceae*		0.2±0	0.3±0	0.2±0	0.3±0	0.3±0b	0.2±0b	0.8±1a
*Acidobacteria*	*Acidobacteria_Gp10*			*Gp10*	2.8±0	4.3±1	2±0	**5.4±1**	1.6±0c	4.4±0a	2.5±1b
	*Acidobacteria_Gp4*			*Gp4*	7.9±1	8.6±1	4.4±0	5±1	7.5±2a	5.8±2a	4.5±1b
	*Acidobacteria_Gp6*			*Gp6*	18.2±2	**22.1±3**	14.2±2	**17.8±0**	13.6±3b	15.8±3a	11.1±2c
*Firmicutes*	*Bacilli*	*Lactobacillales*			0.1±0	0.1±0	0±0	0.4±0	0.1±0b	0.2±0ab	0.5±0a
	*Clostridia*	*Clostridiales*	*Clostridiaceae*	*Clostridium*	0.5±0	0.4±0	1.2±1	1.1±0	1.4±0b	1.7±0b	3.7±1a
	*Clostridia*		*Peptostreptococcaceae*	*Peptostreptococcus*	0.1±0	0.1±0	0.1±0	0.5±0	0.3±0b	0.4±0ab	0.8±0a
*Bacteroidetes*	*Sphingobacteria*	*Sphingobacteriales*	*Cytophagaceae*	*Adhaeribacter*	1.7±0	0.9±0	1.5±0	0.7±0	2.1±1a	0.6±0b	0.4±0b
*Actinobacteria*	*Actinobacteria*	*Actinomycetales*	*Microbacteriaceae*	*Leifsonia*	0±0	0.1±0	0±0	0.1±0	0±0b	0.2±0b	0.4±0a
*Chloroflexi*	*Anaerolineae*	*Anaerolineales*	*Anaerolineaceae*		1±0	1.1±0	1.9±0	1.9±0	1.5±0b	1.8±1ab	2.2±1a
*Gemmatimonadetes*	*Gemmatimonadetes*	*Gemmatimonadales*	*Gemmatimonadaceae*	*Gemmatimonas*	1.1±1	1.6±0	0.5±0	**1.7±2**	0.7±0c	1.6±1b	2.5±1a
*Planctomycetes*	*Planctomycetacia*	*Planctomycetales*	*Planctomycetaceae*		2.7±0	2.6±1	2.5±0	2.4±0	2.6±1a	2.5±0a	1.7±0b
*Verrucomicrobia*	*Subdivision3*			*Subdivision3_genera_incertae_sedis*	0.4±0	0.3±0	0.6±0	0.5±0	0.4±0a	0.5±0a	0.1±0b

Bold numbers indicate taxa with significantly higher relative abundance in SDZ treated or non treated soils; letters a, b and c indicate taxa with significant difference in relative abundance between S0, S10 and S100 treated soils collected at day 193. Soils were collected 60 days after each manure application.

## Discussion

The importance of the soil microbiome for various ecosystem services such as nutrient cycling, soil fertility, degradation of pollutants, and plant growth promotion is well recognized [29]. However, antibiotics introduced into agricultural fields via manure might alter the ability of soil microbes to fulfill crucial ecosystem services, changing the diversity and activity of key functional groups by enhancing antibiotic resistant populations while decreasing the abundance of sensitive populations in soils [16], [30]. In the present study, the effects of repeated application of SDZ manure on soil bacterial communities were explored by barcoded pyrosequencing of 16S rRNA gene amplicons from TC-DNA. More pronounced changes of bacterial communities were observed for soils that were repeatedly treated with SDZ manure. The strongest effects were observed for soils treated three times with manure containing a high concentration of SDZ (Figures 2 and 4). For the first time, taxonomic groups affected by the presence of SDZ were systematically identified, and numerous taxa were found with significantly altered relative abundance (Tables 1 and 2). These findings largely extended our understanding of the influence of SDZ introduced via manure on soil bacterial communities.

Although the bioavailable fraction of SDZ in soil rapidly decreased after each manure application [16], cumulative inputs of SDZ via manure still affected bacterial communities. Also in the study by Byrne-Bailey *et al.* [31], the proportion of sulfachloropyridazine resistant bacteria increased in soils after treatment with manure containing antibiotics. The repeated application of SDZ manure to grassland soil was found to affect nitrite oxidizing bacteria [32]. The influence of SDZ on soil bacterial communities is more likely to be detected when additional nutrients (root exudates, manure) are added to stimulate bacterial growth [33], [34] as SDZ affects only growing bacteria and bacterial activities are generally low in oligotrophic environments such as soils.

The amendment of soil with SDZ manure resulted in a significantly increased relative abundance of numerous Gram-negative and Gram-positive taxa such as *Devosia*, *Shinella*, *Stenotrophomonas*, *Clostridium*, *Peptostreptococcus*, *Leifsonia*, *Gemmatimonas*, suggesting that the presence of SDZ provided a selective advantage for these taxa. Mainly the increase in bacteria affiliated to *Clostridia* detected shortly after the addition of manure likely resulted from manure-derived bacteria. In our study more than 60% of sequences in manure could be affiliated to *Clostridium* (data not shown), which confirms data from previous studies where the dominance of *Clostridium* in manure has been reported [35]–[37]. A steep decrease of *Clostridia* was observed 60 days after the application. Only in S100 soils the relative abundance of *Clostridia* was found to be significantly higher than in the other soils on day 193, suggesting that the high concentration of SDZ slowed down the decline of *Clostridium* populations. Whether the enhanced abundance of *Clostridium* in S100 soils was due to the selection of SDZ resistant populations deserves further research.

Although the resistance to SDZ was not reported yet for most of these taxa, it is likely that some of the populations enriched in the present study might be resistant to SDZ either due to the presence of resistance genes or due to intrinsic resistance. Isolates belonging to *Stenotrophomonas* and *Clostridium* were previously reported to be resistant to other sulfonamide antibiotics [38], [39]. Based on the same TC-DNA, Heuer *et al.* [16] found an accumulation of *sul* gene carrying populations in the soil repeatedly treated with SDZ manure. *Sul* genes located on mobile genetic elements such as class 1 integrons or plasmids might have spread among different taxa [40], [41]. The presence of antibiotics, even at very low concentrations could accelerate the genetic exchanges between bacteria [42], [43]. The enhanced transferability of resistant genes was recently also reported by Jechalke *et al.* [15] in a field experiment using manure from SDZ-treated pigs. However, we cannot exclude that some of the taxa were intrinsically resistant to, or even able to mineralize SDZ as a SDZ degrader identified as *Microbacterium lacus* was recently isolated from the same Merzenhausen soil [44]. Interestingly, in the present study an OTU affiliated to *Leifsonia*, which is phylogenetically closely related to *Microbacterium* was found to be enriched in S100 soils.

In this study, the consensus sequences of an enriched OTU in soils treated with manure shared high similarity with the 16S rRNA gene of *S. maltophilia* which is also known as a human pathogen [38]. However, these enriched bacteria are not necessarily human pathogens as *Stenotrophomonas* was also found in different environments such as rhizosphere or soils [45], [46]. Nevertheless, the enrichments of *Shinella*, *Stenotrophomonas*, *Clostridium* and *Peptostreptococcus* in soil which was continually treated with manure containing a high concentration of SDZ still urge for further investigation due to potential implications for public health. The enrichment of these bacteria, which are phylogenetically closely related to human pathogens, may improve the chance of transferring antibiotic resistance genes to human pathogens, since horizontal gene transfer is more prevalent between closely related organisms than between those distantly related (reviewed by Boto [47]). Soil particles carrying viable bacteria can be transported over long distances and might contribute to the spreading of antibiotic resistant bacteria over wide geographic ranges [48]. The ecological role of taxa such as *Devosia*, *Leifsonia* and *Gemmatimonas* has not been fully understood and thus the effect of their changed abundance on soil functions remains to be explored. Few studies suggested that some members of *Devosia* [49], [50] and *Gemmatimonas* [51] might be associated with nitrogen cycling.

The relative abundances of several other taxa such as *Pseudomonas*, *Lysobacter*, *Hydrogenophaga*, *Haliangium*, and *Adhaeribacter* were found to be significantly lower in the soils treated by SDZ manure. Several members of *Lysobacter* or *Pseudomonas* are known as biological control agents against soilborne phytopathogens such as *Rhizoctonia solani*, *Thielaviopsis basicola* [52], [53]. Several studies suggested that these beneficial bacteria belonging to *Pseudomonas* or *Lysobacter* probably play an important role for soil suppressiveness and plant growth [45], [54]. In the present study, the consensus sequences of an OTU with decreased relative abundance in soil treated with SDZ manure shared 100% similarities with 16S rRNA gene of the strain of *P. brassicacearum* (NCBI accession number: NR_074834) which is a beneficial root-associated bacterium [55]. The decline of *Pseudomonas* and *Lysobacter* in arable soils might increase the susceptibility towards fungal pathogens. However, in general the application of manure to soils is one strategy to suppress soilborne diseases. Our data showed that the relative abundance of *Pseudomonas* was much higher in soils treated with manure than in untreated soils on days 3 and 193. The presence of SDZ in manure likely reduces the beneficial effects of manure on improving soil health as the abundance of *Pseudomonas* was reduced. However, resilience of these beneficial populations might still occur in the rhizosphere as decreased concentrations of SDZ were recently observed in the rhizosphere of maize and grass compared to bulk soil [6]. Little is known about the ecological roles of *Hydrogenophage*, *Haliangium*, *Adhaeribacter* in soil. *Hydrogenophaga* spp. were reported as main biphenyl degraders in the rhizosphere of horseradish (*Armoracia rusticana*) contaminated with polychlorinated biphenyls [56].

The present study is the first to explore the influence of veterinary antibiotics entering soil via manure on the diversity and abundance of *Acidobacteria*. The results revealed that Gp4 was negatively affected by repeated application of manure containing the high concentration of SDZ. Low concentrations of SDZ in manure seemed to favor Gp6 and Gp10, while high concentrations of SDZ could deprive the effects. *Acidobacteria* are known to be adapted to an oligotrophic lifestyle. Indeed, in the present study the relative abundance of *Acidobacteria* was significantly higher in the untreated soil than in the soil amended with manure. Due to their high abundance and ubiquitous distribution in soils, *Acidobacteria* might play an important role in terrestrial ecosystems. In combination with the analysis of three genomes of a*cidobacterial* isolates, Ward *et al.* [57] suggested that *Acidobacteria* might significantly contribute to the terrestrial carbon cycle.

Along with nutrients, a large amount of manure bacteria was also introduced into soil which could have also contributed to the dramatically altered bacterial community compositions observed in the present study shortly after each manure application. However, the majority of these introduced manure bacteria might not be very well adapted to soil, allowing the resilience of the indigenous soil bacteria community. In the present study we showed that repeated application of manure spiked with SDZ to soil increased the degree of disturbance and reduced the resilience of soil bacterial communities compared to a single application of manure spiked with SDZ or soils treated with manure without antibiotics. In a recent study by Poulsen *et al.* [58], the taxonomic composition of bacterial communities based on the abundance of phyla and proteobacterial classes were highly similar between soils with amendments of different organic wastes such as manure. In the present study, we observed clear effects of manure on the soil bacterial community composition as the NMDS analysis revealed separated groups for S0 and untreated soils on day 193 (Figure 3). In contrast to several subgroups of the *Acidobacteria*, *Myxococcales* (*Deltaproteobacteria*), numerous taxa belonging to different phyla (*Proteobacteria, Firmicutes* and *Bacteriodetes*) were observed with significantly higher relative abundance in the soils amended with manure than in the untreated control 60 days after the third application. Very likely the added nutrients favored taxa with a more copiotrophic lifestyle. In contrast to the field experiment analyzed by Poulsen *et al.* [58], the conditions in the present experiment were more controlled and the time period after the last manure amendment might have been shorter although this was not specified in the Poulsen study.

In summary, repeated input of SDZ into the Merzenhausen soil via manure, especially at a high concentration, caused pronounced changes in soil bacterial communities. The presence of SDZ provided a selective advantage for species affiliated to the genera *Devosia, Shinella, Stenotrophomonas, Clostridium, Peptostreptococcus, Leifsonia, Gemmatimonas*, while suppressing *Pseudomonas, Lysobacter, Hydrogenophage, Haliangium, Adhaeribacter*. We could show that SDZ-containing manure caused a more pronounced disturbance of the soil bacterial community compared to control manure and reduced resilience at least for the time period studied. However, whether the findings obtained can be generalized to other soil types with different soil properties remains an open question which needs further attention. Furthermore, mainly the long-term consequences of repeated manure application for soil ecosystem functions and human health demand further investigations.

## Supporting Information

File S1 **Supporting Files.** Table S1. The number of replicates per treatment included in the final sequencing run using the reverse primer. The numbers in brackets indicate the number of samples sequenced using the forward primer. Table S2. Taxa with significantly different relative abundance between soils on day 3 using the reverse primer (Bonferroni adjusted *p* value<0.05). Table S3. Taxa with significantly different relative abundance between soils on day 3 using the forward primer (Bonferroni adjusted *p* value<0.05). Table S4. Relative abundance of detected phyla based on the average of the forward and reverse dataset. Table S5. Taxa with significantly different relative abundance between soils on day 193 using the reverse primer (Bonferroni adjusted *p* value<0.05). Table S6. Taxa with significantly different relative abundance between soils on day 193 using the forward primer (Bonferroni adjusted *p* value<0.05). Table S7. Taxa with significantly different relative abundance between manured soils with or without SDZ treatments as revealed by barcoded pyrosequencing using the forward primer (Bonferroni adjusted *p* value<0.05). Table S8. Taxa with significantly different relative abundance between manured soils with or without SDZ treatments as revealed by barcoded pyrosequencing using the forward primer (Bonferroni adjusted *p* value<0.05). Figure S1. Non metric multidimensional scaling based on OTU reports acquired by the forward primer sequencing. Balls: untreated soils; cubes: S0; octahedron: S10; pyramid: S100.
(DOC)
