# Supplementary material for: Dynamics of Soil Bacterial Communities in Response to Repeated Application of Manure Containing Sulfadiazine
Source: PLoS One. 2014 Mar 26;9(3):e92958. doi: 10.1371/journal.pone.0092958 (PMC3966856; doi:10.1371/journal.pone.0092958)
Supplement: File S1 — Supporting Files. Table S1. The number of replicates per treatment included in the final sequencing run using the reverse primer. The numbers in brackets indicate the number of samples sequenced using the forward primer. Table S2. Taxa with significantly different relative abundance between soils on day 3 using the reverse primer (Bonferroni adjusted p value<0.05). Table S3. Taxa with significantly different relative abundance between soils on day 3 using the forward primer (Bonferroni adjusted p value<0.05). Table S4. Relative abundance of detected phyla based on the average of the forward and reverse dataset. Table S5. Taxa with significantly different relative abundance between soils on day 193 using the reverse primer (Bonferroni adjusted p value<0.05). Table S6. Taxa with significantly different relative abundance between soils on day 193 using the forward primer (Bonferroni adjusted p value<0.05). Table S7. Taxa with significantly different relative abundance between manured soils with or without SDZ treatments as revealed by barcoded pyrosequencing using the forward primer (Bonferroni adjusted p value<0.05). Table S8. Taxa with significantly different relative abundance between manured soils with or without SDZ treatments as revealed by barcoded pyrosequencing using the forward primer (Bonferroni adjusted p value<0.05). Figure S1. Non metric multidimensional scaling based on OTU reports acquired by the forward primer sequencing. Balls: untreated soils; cubes: S0; octahedron: S10; pyramid: S100. (DOC) [file pone.0092958.s001.doc]

**Supporting Information**

**Dynamics of soil bacterial communities in response to repeated application of manure containing sulfadiazine**

Guo-Chun Ding^1^, Viviane Radl^2^, Brigitte Schloter-Hai^2^, Sven Jechalke^1^, Holger Heuer^1^, Kornelia Smalla^1^*, Michael Schloter^2^

^1^ Julius Kühn-Institut - Federal Research Centre for Cultivated Plants (JKI), Institute for Epidemiology and Pathogen Diagnostics, Messeweg 11-12, D-38104 Braunschweig, Germany

^2^ Helmholtz Zentrum München, German Research Center for Environmental Health, Research Unit for Environmental Genomics; Ingolstädter Landstr. 1, D-85764 Neuherberg, Germany

**Number of Tables: 8**

**Number of Figures: 1**

^*^for correspondence: Email: [kornelia.smalla@jki.bund.de](mailto:kornelia.smalla@jki.bund.de); Tel.: (+49) 531 2993814; Fax: (+49) 531 2993006

**Table S1** The number of replicates per treatment included in the final sequencing run using the reverse primer. The numbers in brackets indicate the number of samples sequenced using the forward primer.

|  | Treatment | |  |  |  |
| --- | --- | --- | --- | --- | --- |
| day | U | S0 | S10 | S100 |  |
| 3 | 3 | 3 | 3 | - |  |
| 60 | - | 3 | 3 | - |  |
| 66 | - | 3 | 3 | - |  |
| 123 | - | 2 | 2 | - |  |
| 136 | - | 3 | 3 | - |  |
| 193 | 4(5) | 5 | 5 | 4 | Total |
| Sum | 7(8) | 19 | 19 | 4 | 49(50) |

**Table S2 Taxa with significantly different relative abundance between soils at day 3 using the reverse primer (Bonferroni adjusted *p* value<0.05).**

| Phylum | Class | Order | Family | Genus | Day3 U | Day3 S0 | Day3 S10 |
| --- | --- | --- | --- | --- | --- | --- | --- |
| *Proteobacteria* | *Alphaproteobacteria* | *Rhizobiales* | *Rhizobiaceae* |  | 0.4±0b | 0.6±0ab | 1±0a |
|  |  | *Sphingomonadales* | *Sphingomonadaceae* |  | 4.6±0ab | 3.8±1b | 5.2±3a |
|  | *Betaproteobacteria* | *Burkholderiales* | *Burkholderiales_incertae_sedis* | *Methylibium* | 1.5±0a | 0.6±0b | 1±0ab |
|  |  | *Burkholderiales* | *Comamonadaceae* | *Delftia* | 0±0b | 1.1±2a | 0±0ab |
|  | *Gammaproteobacteria* | *Pseudomonadales* | *Pseudomonadaceae* | *Pseudomonas* | 0±0b | 0.8±1a | 0.2±0b |
|  |  | *Xanthomonadales* | *Xanthomonadaceae* | *Lysobacter* | 2±1ab | 2.1±1a | 1.2±0b |
|  |  |  |  | *Pseudoxanthomonas* | 0.2±0b | 0.8±1a | 0.7±0ab |
|  |  |  |  | *Stenotrophomonas* | 0.1±0b | 1.6±2a | 0.1±0b |
|  | *Deltaproteobacteria* | *Myxococcales* |  |  | 1.8±0a | 1.1±0b | 1.2±0ab |
| *Firmicutes* | *Bacilli* | *Bacillales* | *Bacillaceae* | *Bacillus* | 0.5±0b | 4.9±1a | 5.2±2a |
|  |  |  | *Planococcaceae* |  | 0±0b | 0.5±0a | 0.5±0a |
|  |  | *Lactobacillales* | *Lactobacillaceae* | *Lactobacillus* | 0±0b | 3.2±0a | 2.9±2a |
|  |  | *Lactobacillales* | *Streptococcaceae* | *Streptococcus* | 0±0b | 2.1±1a | 2.3±0a |
|  | *Clostridia* | *Clostridiales* | *Clostridiaceae* | *Clostridium* | 0.1±0b | 1.6±1a | 1.8±0a |
|  |  |  | *Lachnospiraceae* |  | 0±0b | 1.1±0a | 1.2±0a |
|  |  |  | *Ruminococcaceae* |  | 0±0b | 1.2±0a | 1.3±0a |
|  | *Erysipelotrichi* | *Erysipelotrichales* | *Erysipelotrichaceae* |  | 0±0b | 1.2±0a | 1.4±1a |
| *Acidobacteria* | *Acidobacteria_Gp10* |  |  | *Gp10* | 2.8±0a | 1±0b | 1.5±0b |
|  | *Acidobacteria_Gp4* |  |  | *Gp4* | 6.9±1a | 3.3±0c | 4.7±1b |
|  | *Acidobacteria_Gp6* |  |  | *Gp6* | 18.4±2a | 8.7±1b | 10.5±2b |
|  | *Acidobacteria_Gp7* |  |  | *Gp7* | 1.7±0a | 0.6±0b | 0.8±0b |
| *Actinobacteria* | *Actinobacteria* | *Actinomycetales* | *Micrococcaceae* | *Arthrobacter* | 0.1±0b | 2.2±0a | 1.5±0a |
|  |  | *Solirubrobacterales* |  |  | 1.2±1a | 0.7±0ab | 0.6±0b |
| *Bacteroidetes* | *Sphingobacteria* | *Sphingobacteriales* | *Cytophagaceae* | *Adhaeribacter* | 1.5±0a | 1.5±0a | 0.7±0b |
| *Gemmatimonadetes* | *Gemmatimonadetes* |  |  |  | 0.6±0a | 0.2±0b | 0.3±0ab |
| *Planctomycetes* | *Planctomycetacia* | *Planctomycetales* | *Planctomycetaceae* |  | 3.8±0a | 2±0b | 1.7±0b |

U: untreated soils; S0: soil treated with SDZ free manure; S10: soil treated with manure containing 10 mg/kg SDZ; S100: soil treated with manure containing 100 mg/kg SDZ; letters a, b and c indicate taxa with significant difference in relative abundance;

**Table S3 Taxa with significantly different relative abundance between soils at day 3 using the forward primer (Bonferroni adjusted *p* value<0.05).**

| Phylum | Class | | Order | Family | Genus | Day3 U | Day3 S0 | Day3 S10 |
| --- | --- | --- | --- | --- | --- | --- | --- | --- |
| *Proteobacteria* | *Alphaproteobacteria* | | *Rhizobiales* | *Hyphomicrobiaceae* |  | 1.1±0a | 0.5±0b | 0.8±0ab |
|  | *Betaproteobacteria* | | *Burkholderiales* | *Alcaligenaceae* |  | 0±0b | 0.5±0a | 0.3±0ab |
|  |  | |  | *Burkholderiales_incertae_sedis* | | 1.6±0a | 0.7±0b | 1±1ab |
|  |  | |  | *Comamonadaceae* | *Delftia* | 0±0b | 1±2a | 0±0ab |
|  | *Deltaproteobacteria* | | *Myxococcales* |  |  | 1.6±0a | 0.9±0b | 0.9±0ab |
|  | *Gammaproteobacteria* | | *Pseudomonadales* | *Pseudomonadaceae* | *Pseudomonas* | 0.1±0b | 0.9±1a | 0.1±0b |
|  |  | | *Xanthomonadales* | *Xanthomonadaceae* | *Lysobacter* | 0.4±0b | 0.8±1a | 0.5±0ab |
|  |  | |  |  | *Stenotrophomonas* | 0.1±0b | 1.3±2a | 0.1±0b |
| *Firmicutes* | *Bacilli* | | *Bacillales* | *Bacillaceae* | *Bacillus* | 0.4±0b | 3.3±1a | 3.7±1a |
|  |  | |  | *Planococcaceae* | *Paenisporosarcina* | 0.1±0b | 1.6±0a | 2.2±1a |
|  |  | | *Lactobacillales* | *Lactobacillaceae* | *Lactobacillus* | 0±0b | 3.3±1a | 3±2a |
|  |  | |  | *Streptococcaceae* | *Streptococcus* | 0±0b | 1.9±1a | 2.2±1a |
|  | *Clostridia* | | *Clostridiales* | *Clostridiaceae* | *Clostridium* | 0.1±0b | 1.3±1a | 1.5±0a |
|  |  | |  | *Ruminococcaceae* |  | 0±0b | 1.3±0a | 1.6±0a |
|  | *Erysipelotrichi* | | *Erysipelotrichales* | *Erysipelotrichaceae* |  | 0±0b | 1.1±0a | 1.1±0a |
| *Acidobacteria* | *Acidobacteria_Gp10* | | |  | *Gp10* | 3.1±1a | 1.1±0b | 1.3±1b |
|  | *Acidobacteria_Gp4* | | |  | *Gp4* | 6.7±1a | 3.4±1b | 3.9±1b |
|  | *Acidobacteria_Gp6* | | |  | *Gp6* | 15.7±2a | 7.3±0b | 9±2b |
|  | *Acidobacteria_Gp7* | | |  | *Gp7* | 1.7±0a | 0.5±0b | 0.9±0ab |
| *Actinobacteria* | *Actinobacteria* | *Actinomycetales* | | *Micrococcaceae* | *Arthrobacter* | 0.2±0b | 2.2±0a | 1.5±0a |
| *Gemmatimonadetes* | *Gemmatimonadetes* | | |  |  | 0.5±0a | 0.1±0b | 0.1±0ab |
| *Planctomycetes* | *Planctomycetacia* | *Planctomycetales* | | *Planctomycetaceae* |  | 4±0a | 1.9±0b | 1.2±0b |

U: untreated soils; S0: soil treated with SDZ free manure; S10: soil treated with manure containing 10 mg/kg SDZ; S100: soil treated with manure containing 100 mg/kg SDZ; letters a, b and c indicate taxa with significant difference in relative abundance;

**Table S4** Relative abundance of detected phyla based on the average of the forward and reverse dataset.

|  | Day 3 | | | Day 60 | | Day 66 | | Day 123 | | Day 136 | | Day 193 | | | |
| --- | --- | --- | --- | --- | --- | --- | --- | --- | --- | --- | --- | --- | --- | --- | --- |
|  | U | S0 | S10 | S0 | S10 | S0 | S10 | S0 | S10 | S0 | S10 | U | S0 | S10 | S100 |
| *Proteobacteria* | 32.4 ± 1.2 | 34.1 ± 5.1 | 30.1 ± 2.4 | 36.6 ± 2.1 | 29.1 ± 0.7 | 43 ± 2.2 | 27.3 ± 1.2 | 45.5 ± 0 | 32.6 ± 0.4 | 45.8 ± 5.5 | 35.5 ± 4.5 | 27.7 ± 2 | 41.9 ± 5.8 | 34.5 ± 2.7 | 39.3 ± 1 |
| *Acidobacteria* | 30.9 ± 3.3 | 14 ± 0.6 | 17.8 ± 4.2 | 30.8 ± 2.8 | 37.5 ± 1.4 | 20 ± 2.9 | 27 ± 2.4 | 22.4 ± 0 | 30.8 ± 1.3 | 15.2 ± 6.2 | 16.8 ± 3.9 | 33.7 ± 3.8 | 24.5 ± 5.4 | 28.2 ± 4 | 20.1 ±2.8 |
| *Firmicutes* | 1.5 ± 0.2 | 25.3 ± 6.2 | 25.8 ± 6.5 | 1.7 ± 0.2 | 1.9 ± 0.5 | 15.8 ± 1.7 | 20.1 ± 2.7 | 2.8 ± 0.1 | 3.4 ± 0.4 | 13.1 ± 3 | 17.9 ± 2.3 | 1.5 ± 0.3 | 3.1 ± 0.5 | 3.9 ± 0.9 | 7.4 ± 1.4 |
| *Actinobacteria* | 6.4 ± 2.9 | 8.9 ± 0.8 | 7.5 ± 2 | 6.9 ± 2.7 | 6.2 ± 2.3 | 3.7 ± 1.7 | 4.7 ± 0.9 | 3.3 ± 0.3 | 3.8 ± 0.2 | 6.2 ± 6.2 | 7.5 ± 7 | 4.8 ± 4 | 3.7 ± 2.2 | 4.2 ± 2.1 | 3.4 ± 0.8 |
| *Chloroflexi* | 3.6 ± 0.8 | 2.9 ± 0.7 | 3.1 ± 0.2 | 2.7 ± 0.4 | 3.8 ± 0.2 | 1.9 ± 0.7 | 3.6 ± 1.3 | 5.6 ± 0.1 | 6.7 ± 0.1 | 4.1 ± 0.6 | 5.5 ± 1 | 4.4 ± 0.5 | 4.2 ± 0.6 | 5.3 ± 0.9 | 9.4 ± 2.1 |
| *Planctomycetes* | 3.9 ± 0.3 | 1.9 ± 0.2 | 1.5 ± 0.2 | 2.7 ± 0.3 | 2.6 ± 0.6 | 1.9 ± 0.6 | 2 ± 0.5 | 2.6 ± 0 | 2.4 ± 0.1 | 1.5 ± 0.7 | 1.7 ± 0.3 | 2.7 ± 0.3 | 2.5 ± 0.5 | 2.5 ± 0.4 | 1.8 ± 0.3 |
| *Bacteroidetes* | 1.9 ± 0.5 | 2.2 ± 0.3 | 1.5 ± 0.3 | 2 ± 0.9 | 1 ± 0.3 | 2.5 ± 1.3 | 0.7 ± 0.2 | 1.6 ± 0.1 | 0.7 ± 0.1 | 1.7 ± 0.4 | 0.6 ± 0.2 | 1.1 ± 0.3 | 2.6 ± 1.9 | 0.6 ± 0.2 | 0.5 ± 0.1 |
| *Verrucomicrobia* | 0.8 ± 0.2 | 0.7 ± 0.2 | 0.7 ± 0.1 | 1.2 ± 0.4 | 1 ± 0.4 | 0.7 ± 0.4 | 0.8 ± 0.5 | 1.5 ± 0 | 1.4 ± 0.1 | 1.1 ± 0.1 | 0.8 ± 0.3 | 1 ± 0.4 | 1.5 ± 0.2 | 1.6 ± 0.2 | 0.9 ± 0.4 |
| *Gemmatimonadetes* | 0.5 ± 0.3 | 0.2 ± 0.1 | 0.2 ± 0.1 | 1 ± 0.7 | 1.4 ± 0.6 | 0.3 ± 0.1 | 0.6 ± 0.2 | 0.5 ± 0.1 | 1.6 ± 0.3 | 0.4 ± 0.2 | 0.9 ± 0.7 | 0.7 ± 0.4 | 0.7 ± 0.6 | 1.4 ± 1.2 | 2.2 ± 1 |
| *OP10* | 0.3 ± 0.1 | 0.1 ± 0 | 0.3 ± 0.1 | 0.3 ± 0 | 0.4 ± 0 | 0.1 ± 0 | 0.2 ± 0.2 | 0.4 ± 0.1 | 0.4 ± 0 | 0.3 ± 0 | 0.4 ± 0.1 | 0.6 ± 0.3 | 0.5 ± 0.1 | 0.5 ± 0.1 | 0.8 ± 0.3 |
| *Nitrospira* | 0.3 ± 0 | 0.2 ± 0.1 | 0.2 ± 0.1 | 0.4 ± 0 | 0.4 ± 0.1 | 0.2 ± 0.1 | 0.3 ± 0.2 | 0.2 ± 0 | 0.3 ± 0 | 0.2 ± 0.1 | 0.2 ± 0.1 | 0.3 ± 0.2 | 0.4 ± 0 | 0.5 ± 0.1 | 0.2 ± 0.1 |
| *Deinococcus-Thermus* | 0 ± 0 | 0.2 ± 0.2 | 0.1 ± 0.1 | 0 ± 0 | 0 ± 0 | 0.1 ± 0.1 | 0.2 ± 0.1 | 0.1 ± 0 | 0.1 ± 0 | 0.1 ± 0 | 0.1 ± 0.1 | 0 ± 0 | 0.1 ± 0.1 | 0.1 ± 0.1 | 0.1 ± 0 |
| *Chlamydiae* | 0 ± 0 | 0 ± 0 | 0 ± 0 | 0 ± 0 | 0.1 ± 0 | 0 ± 0 | 0 ± 0 | 0.1 ± 0 | 0.1 ± 0 | 0 ± 0 | 0 ± 0 | 0.1 ± 0 | 0.1 ± 0.1 | 0.1 ± 0.1 | 0 ± 0.1 |
| *Cyanobacteria* | 0 ± 0 | 0.2 ± 0.1 | 0.1 ± 0.1 | 0 ± 0 | 0 ± 0 | 0 ± 0 | 0 ± 0 | 0 ± 0 | 0 ± 0 | 0 ± 0 | 0 ± 0 | 0.1 ± 0 | 0 ± 0 | 0 ± 0 | 0 ± 0 |

U: untreated soils; S0: soil treated with SDZ free manure; S10: soil treated with manure containing 10 mg/kg SDZ; S100: soil treated with manure containing 100 mg/kg SDZ;

**Table S5 Taxa with significantly different relative abundance between soils at day 193 using the reverse primer (Bonferroni adjusted *p* value<0.05).**

| Phylum | Class | Order | Family | Genus | Day 193 U | Day 193 S0 | Day 193 S10 | Day 193 S100 |
| --- | --- | --- | --- | --- | --- | --- | --- | --- |
| *Proteobacteria* | *Alphaproteobacteria* | *Caulobacterales* | *Caulobacteraceae* |  | 0.2±0b | 0.5±0a | 0.5±0ab | 0.4±0ab |
|  |  | *Rhizobiales* | *Hyphomicrobiaceae* | *Devosia* | 0.1±0c | 1.5±1b | 2.2±1ab | 3±1a |
|  |  | *Rhodospirillales* |  |  | 1.1±0b | 1.8±0a | 1.6±1ab | 1.1±0b |
|  |  | *Sphingomonadales* | *Erythrobacteraceae* | | 0.1±0b | 0.4±0a | 0.1±0ab | 0.1±0ab |
|  |  |  | *Sphingomonadaceae* | *Sphingomonas* | 1.1±0c | 3.8±0a | 1.8±0bc | 2.3±0b |
|  | *Betaproteobacteria* | *Burkholderiales* | *Alcaligenaceae* |  | 0±0b | 0.1±0b | 0.2±0ab | 0.5±0a |
|  |  |  | *Burkholderiales_incertae_sedis* | *Methylibium* | 0.4±0b | 0.9±0a | 0.8±1a | 0.4±0ab |
|  |  |  | *Comamonadaceae* | *Hydrogenophaga* | 0±0b | 0.5±0a | 0.1±0ab | 0±0ab |
|  |  |  |  | *Simplicispira* | 0±0b | 0.1±0b | 0.2±0b | 1.7±3a |
|  |  |  | *Oxalobacteraceae* |  | 0.9±0b | 2±1a | 0.5±0bc | 0.3±0c |
|  |  | *Methylophilales* | *Methylophilaceae* |  | 0.3±0ab | 0.5±0a | 0.3±0ab | 0±0b |
|  |  | *Rhodocyclales* | *Rhodocyclaceae* | *Shinella* | 0±0b | 0±0b | 0.4±0b | 1.4±1a |
|  | *Deltaproteobacteria* | *Myxococcales* |  |  | 4.4±1a | 3.5±1b | 1.1±0c | 1.2±1c |
|  | *Gammaproteobacteria* | *Legionellales* | *Coxiellaceae* |  | 0±0b | 0.1±0ab | 0.3±0a | 0.3±0ab |
|  |  | *Pseudomonadales* | *Pseudomonadaceae* | *Pseudomonas* | 0±0b | 1±2a | 0±0b | 0±0b |
|  |  | *Xanthomonadales* | *Xanthomonadaceae* | *Arenimonas* | 0.1±0ab | 0.4±0a | 0.1±0ab | 0.1±0b |
|  |  |  |  | *Pseudoxanthomonas* | 0.1±0c | 0.6±0b | 0.4±0b | 1.8±1a |
|  |  |  |  | *Rhodanobacter* | 0±0b | 0.3±0b | 0.6±1a | 0.1±0b |
|  |  |  |  | *Stenotrophomonas* | 0±0bc | 0±0c | 0.3±0b | 3.1±2a |
| *Acidobacteria* | *Acidobacteria_Gp10* |  |  | *Gp10* | 2.7±1bc | 2.2±1c | 6.6±1a | 3.6±1b |
|  | *Acidobacteria_Gp4* |  |  | *Gp4* | 9±1a | 8.1±3ab | 6.1±1b | 5.3±1c |
|  | *Acidobacteria_Gp5* |  |  | *Gp5* | 1.3±1a | 0.5±0b | 0.5±0b | 0.4±0b |
|  | *Acidobacteria_Gp6* |  |  | *Gp6* | 15.6±2a | 11±2bc | 12.4±3b | 9±1c |
|  | *Acidobacteria_Gp7* |  |  | *Gp7* | 2.3±0a | 0.9±0b | 1.1±0b | 0.8±0b |
| *Firmicutes* | *Bacilli* | *Bacillales* |  |  | 1.3±0a | 0.9±0ab | 0.7±0b | 0.7±0b |
|  |  | *Lactobacillales* |  |  | 0±0bc | 0.1±0c | 0.4±0ab | 0.6±0a |
|  | *Clostridia* | *Clostridiales* | *Clostridiaceae* | *Clostridium* | 0.1±0c | 1.3±0b | 1.7±1b | 4.3±1a |
|  |  |  | *Peptostreptococcaceae* | *Peptostreptococcus* | 0±0c | 0.4±0b | 0.5±0ab | 1.1±0a |
| *Actinobacteria* | *Actinobacteria* | *Actinomycetales* | *Microbacteriaceae* | *Leifsonia* | 0.1±0b | 0.1±0b | 0.1±0b | 0.5±0a |
|  |  | *Solirubrobacterales* |  |  | 0.5±0a | 0.2±0b | 0.3±0ab | 0.2±0ab |
| *Bacteroidetes* | *Sphingobacteria* | *Sphingobacteriales* | *Cytophagaceae* | *Adhaeribacter* | 0.9±0b | 1.9±1a | 0.4±0bc | 0.2±0c |
| *Chloroflexi* |  |  |  |  | 4.2±0b | 4±1b | 4.8±1b | 8.5±2a |
| *Gemmatimonadetes* | *Gemmatimonadetes* | *Gemmatimonadales* | *Gemmatimonadaceae* | *Gemmatimonas* | 0.8±0bc | 0.7±1c | 1.2±1b | 1.9±1a |
| *OP10* |  |  |  | *OP10_genera_incertae_sedis* | 0.7±0ab | 0.5±0b | 0.7±0ab | 1±0a |
| *Planctomycetes* | *Planctomycetacia* | *Planctomycetales* | *Planctomycetaceae* |  | 2.6±0a | 2.4±0ab | 2.6±0a | 1.8±0b |

**Table S6 Taxa with significantly different relative abundance between soils at day 193 using the forward primer (Bonferroni adjusted *p* value<0.05).**

| Phylum | Class | Order | Family | Genus | Day 193  U | Day 193  S0 | Day 193  S10 | Day 193 S100 |
| --- | --- | --- | --- | --- | --- | --- | --- | --- |
| *Proteobacteria* | *Alphaproteobacteria* | *Caulobacterales* | *Caulobacteraceae* |  | 0.2±0b | 0.5±0a | 0.5±0ab | 0.4±0ab |
|  |  | *Rhizobiales* | *Hyphomicrobiaceae* | *Devosia* | 0.1±0c | 1.5±0b | 2.6±1a | 3.5±1a |
|  |  |  | *Rhizobiaceae* |  | 0.3±0b | 0.6±0a | 0.4±0ab | 0.4±0b |
|  |  | *Rhodospirillales* | *Rhodospirillaceae* | *Magnetospirillum* | 0.4±0b | 0.8±0ab | 0.9±0a | 0.5±0b |
|  |  | *Sphingomonadales* | *Sphingomonadaceae* | *Novosphingobium* | 0.1±0b | 0.4±0ab | 0.6±0a | 0.4±0a |
|  | *Betaproteobacteria* | *Burkholderiales* | *Alcaligenaceae* |  | 0±0b | 0.1±0b | 0.1±0b | 0.8±0a |
|  |  |  | *Burkholderiales_incertae_sedis* | *Methylibium* | 0.8±0b | 2±1a | 1.8±1a | 0.9±0b |
|  |  |  | *Comamonadaceae* | *Hydrogenophaga* | 0±0b | 0.5±0a | 0.1±0b | 0±0b |
|  |  |  |  | *Variovorax* | 0.2±0b | 0.5±0ab | 0.6±0a | 0.3±0ab |
|  |  |  | *Oxalobacteraceae* | *Herminiimonas* | 0±0ab | 0.5±0a | 0±0ab | 0±0b |
|  |  | *Methylophilales* | *Methylophilaceae* |  | 0.2±0ab | 0.3±0a | 0.1±0ab | 0±0b |
|  |  | *Rhodocyclales* | *Rhodocyclaceae* | *Shinella* | 0±0b | 0±0b | 0.2±0b | 1.2±1a |
|  | *Deltaproteobacteria* | *Myxococcales* | *Haliangiaceae* | *Haliangium* | 1.6±1a | 0.8±0b | 0.1±0c | 0.1±0c |
|  |  |  | *Nannocystaceae* |  | 0.2±0ab | 0.3±0a | 0.2±0ab | 0±0b |
|  |  |  | *Polyangiaceae* |  | 0.3±0b | 0.3±0b | 0.2±0b | 0.8±1a |
|  | *Gammaproteobacteria* | *Legionellales* | *Coxiellaceae* | *Aquicella* | 0±0b | 0.2±0ab | 0.4±0a | 0.3±0a |
|  |  | *Pseudomonadales* | *Pseudomonadaceae* | *Pseudomonas* | 0±0b | 1.2±2a | 0.2±0b | 0±0b |
|  |  | *Xanthomonadales* | *Xanthomonadaceae* | *Lysobacter* | 0.7±0a | 0.9±0a | 0.2±0b | 0.1±0b |
|  |  |  |  | *Pseudoxanthomonas* | 0.1±0c | 0.7±0b | 0.4±0b | 2±2*a |
|  |  |  |  | *Rhodanobacter* | 0±0b | 0.3±0b | 0.9±1a | 0.2±0b |
|  |  |  |  | *Stenotrophomonas* | 0±0b | 0±0b | 0.2±0b | 3.5±3a |
| *Acidobacteria* | *Acidobacteria_Gp10* |  |  | *Gp10* | 2.3±1bc | 1.6±0c | 4.4±0a | 2.5±1b |
|  | *Acidobacteria_Gp25* |  |  | *Gp25* | 0.2±0a | 0.1±0b | 0±0ab | 0.1±0ab |
|  | *Acidobacteria_Gp4* |  |  | *Gp4* | 8.7±2a | 7.5±2ab | 5.8±2b | 4.5±1c |
|  | *Acidobacteria_Gp5* |  |  | *Gp5* | 1.4±0a | 0.6±0b | 0.6±0b | 0.4±0*b |
|  | *Acidobacteria_Gp6* |  |  | *Gp6* | 19.1±1a | 13.6±3c | 15.8±3b | 11.1±2d |
|  | *Acidobacteria_Gp7* |  |  | *Gp7* | 2±0a | 0.9±0b | 0.8±0b | 0.5±0b |
| *Firmicutes* | *Bacilli* | *Bacillales* |  |  | 1.1±0a | 0.8±0ab | 0.7±0b | 0.7±0b |
|  |  | *Lactobacillales* |  |  | 0±0b | 0.1±0b | 0.2±0ab | 0.5±0a |
|  | *Clostridia* | *Clostridiales* | *Clostridiaceae* | *Clostridium* | 0.2±0c | 1.4±0b | 1.7±0b | 3.7±1a |
|  |  | *Clostridiales* | *Peptostreptococcaceae* | *Peptostreptococcus* | 0±0b | 0.3±0b | 0.4±0ab | 0.8±0a |
|  | *Erysipelotrichi* |  |  |  | 0±0b | 0.2±0ab | 0.1±0ab | 0.5±0a |
| *Actinobacteria* | *Actinobacteria* | *Actinomycetales* | *Microbacteriaceae* | *Leifsonia* | 0±0b | 0±0b | 0.2±0b | 0.4±0a |
|  |  |  | *Nocardioidaceae* |  | 0.7±1a | 0.6±0ab | 0.5±0ab | 0.4±0b |
|  |  | *Solirubrobacterales* |  |  | 0.6±0a | 0.2±0b | 0.3±0ab | 0.3±0ab |
| *Bacteroidetes* | *Sphingobacteria* | *Sphingobacteriales* | *Cytophagaceae* | *Adhaeribacter* | 0.9±0b | 2.1±1a | 0.6±0bc | 0.4±0c |
| *Chloroflexi* | *Anaerolineae* | *Anaerolineales* | *Anaerolineaceae* |  | 2.1±0ab | 1.5±0b | 1.8±1ab | 2.2±1a |
| *Gemmatimonadetes* | *Gemmatimonadetes* | *Gemmatimonadales* | *Gemmatimonadaceae* | *Gemmatimonas* | 0.8±0bc | 0.7±0c | 1.6±1b | 2.5±1a |
| *Nitrospira* |  |  |  |  | 0.4±0ab | 0.4±0a | 0.5±0ab | 0.2±0b |
| *Planctomycetes* | *Planctomycetacia* | *Planctomycetales* | *Planctomycetaceae* |  | 2.7±0a | 2.6±1a | 2.5±0a | 1.7±0b |

Table S7 Taxa with significantly different relative abundance between manured soils with or without SDZ treatments as revealed by barcoded pyrosequencing using the forward primer (Bonferroni adjusted *p* value <0.05).

| Phylum | Class | Order | Family | Genus | Day 3 | |  | Day 66 | |  | Day 136 | |
| --- | --- | --- | --- | --- | --- | --- | --- | --- | --- | --- | --- | --- |
|  |  |  |  |  | S0 | S10 |  | S0 | S10 |  | S0 | S10 |
| *Proteobacteria* | *Alphaproteobacteria* | *Rhizobiales* | *Hyphomicrobiaceae* | *Devosia* | 0.1±0 | 0.3±0 |  | 0.5±0 | 1.2±0 |  | 0.9±0 | **2.2±1** |
|  |  | *Sphingomonadales* | *Sphingomonadaceae* | *Sphingomonas* | 3.1±2 | 3.8±2 |  | **5±2** | 2.5±0 |  | **5±1** | 3.1±1 |
|  | *Betaproteobacteria* | *Burkholderiales* | *Burkholderiales_*  *incertae_sedis* |  | 0.7±0 | 1±1 |  | 1.6±0 | 1.2±0 |  | **2.5±1** | 1±0 |
|  |  |  | *Comamonadaceae* |  | **3.1±2** | 1.6±0 |  | 2.3±1 | 1.7±0 |  | 2.1±0 | 2.3±1 |
|  |  |  | *Oxalobacteraceae* |  | 2.4±0 | 2.2±1 |  | **2.5±1** | 0.7±0 |  | **6.1±4** | 0.6±0 |
|  | *Deltaproteobacteria* | *Myxococcales* |  |  | 0.9±0 | 0.9±0 |  | 1.3±0 | 1±0 |  | **2.5±1** | 1.1±0 |
|  | *Gammaproteobacteria* | *Pseudomonadales* | *Pseudomonadaceae* | *Pseudomonas* | **0.9±1** | 0.1±0 |  | **2.4±4** | 0.1±0 |  | 0.3±0 | 0±0 |
|  |  | *Xanthomonadales* | *Xanthomonadaceae* | *Arenimonas* | 0.8±1 | 0.5±0 |  | 1.7±2 | 0.5±0 |  | **1.4±1** | 0.4±0 |
|  |  |  |  | *Lysobacter* | 0.8±1 | 0.5±0 |  | 0.8±0 | 0.2±0 |  | **1.5±1** | 0.2±0 |
|  |  |  |  | *Stenotrophomonas* | **1.3±2** | 0.1±0 |  | 0±0 | 0±0 |  | 0.1±0 | 0.4±0 |
| *Acidobacteria* | *Acidobacteria_Gp10* |  |  | *Gp10* | 1.1±0 | 1.3±1 |  | **1.6±0** | 4.1±0 |  | 1.2±1 | **3.6±0** |
|  | *Acidobacteria_Gp6* |  |  | *Gp6* | 7.3±0 | 9±2 |  | 10.6±2 | **13.6±1** |  | 7.3±3 | 6.9±2 |
| *Firmicutes* | *Bacilli* | *Lactobacillales* |  |  | 5.3±1 | 5.2±1 |  | 3.5±1 | 4.9±1 |  | 2.4±1 | **4.3±1** |
|  | *Clostridia* | *Clostridiales* |  |  | 5.7±2 | 6.3±1 |  | 5.9±1 | 8.5±1 |  | 7.1±1 | **10.2±2** |
| *Actinobacteria* | *Actinobacteria* | *Actinomycetales* |  |  | 8.1±1 | 6.3±2 |  | 2.9±2 | 3.9±1 |  | 5.2±5 | **6.4±6** |
| *Bacteroidetes* | *Sphingobacteria* | *Sphingobacteriales* | *Cytophagaceae* | *Adhaeribacter* | 1.1±0 | 0.8±0 |  | 1.7±1 | 0.5±0 |  | **1.3±0** | 0.4±0 |
| *Chloroflexi* |  |  |  |  | 2.4±1 | 2.7±0 |  | 1.9±1 | **3.5±1** |  | 3.8±1 | 4.8±1 |

Bold numbers indicate taxa with significantly higher relative abundance in SDZ treated or untreated soils. Soils were collected three days after each manure application.

**Table S8** Taxa with significantly different relative abundance between manured soils with or without SDZ treatments as revealed by barcoded pyrosequencing using the forward primer (Bonferroni adjusted *p* value <0.05).

| Phylum | Class | Order | Family | Genus | Day 60 | |  | Day 123 | |  | Day 193 | | |
| --- | --- | --- | --- | --- | --- | --- | --- | --- | --- | --- | --- | --- | --- |
|  |  |  |  |  | S0 | S10 |  | S0 | S10 |  | S0 | S10 | S100 |
| *Proteobacteria* | *Alphaproteobacteria* | *Rhizobiales* | *Hyphomicrobiaceae* | *Devosia* | 0.4±0 | 1.1±0 |  | 1.3±0 | 1.8±0 |  | 1.5±1b | 2.2±1ab | 3±1a |
|  |  | *Rhodospirillales* |  |  | 1±0 | 1.2±0 |  | 1.4±0 | 1.3±0 |  | 1.8±0a | 1.6±1ab | 1.1±0b |
|  |  | *Sphingomonadales* | *Sphingomonadaceae* | *Sphingomonas* | 2.4±0 | 1.9±1 |  | 2.6±1 | 1.8±0 |  | 3.8±0a | 1.8±0b | 2.3±0b |
|  | *Betaproteobacteria* | *Burkholderiales* | *Alcaligenaceae* |  | 0.1±0 | 0.1±0 |  | 0.1±0 | 0.1±0 |  | 0.1±0b | 0.2±0ab | 0.5±0a |
|  |  |  | *Burkholderiales* | *Methylibium* | 1.1±0 | 0.8±0 |  | 0.6±0 | 0.7±0 |  | 0.9±0a | 0.8±1ab | 0.4±0b |
|  |  |  | *Comamonadaceae* | *Hydrogenophaga* | 0.3±0 | 0.1±0 |  | **0.9±0** | 0.1±0 |  | 0.5±0 | 0.1±0 | 0±0 |
|  |  |  |  | *Simplicispira* | 0±0 | 0±0 |  | 0.1±0 | 0.1±0 |  | 0.1±0b | 0.2±0b | 1.7±3a |
|  |  |  | *Oxalobacteraceae* |  | **2.2±0** | 0.8±0 |  | **2.8±0** | 0.5±0 |  | 2±1a | 0.5±0b | 0.3±0b |
|  |  | *Methylophilales* | *Methylophilaceae* |  | 0.1±0 | 0.1±0 |  | 0.6±0 | 0.3±0 |  | 0.5±0a | 0.3±0ab | 0±0b |
|  |  | *Rhodocyclales* | *Rhodocyclaceae* | *Shinella* | 0±0 | 0±0 |  | 0±0 | 0.2±0 |  | 0±0b | 0.4±0b | 1.4±1a |
|  | *Gammaproteobacteria* | *Pseudomonadales* | *Pseudomonadaceae* | *Pseudomonas* | 0.6±1 | 0.2±0 |  | 0.1±0 | 0±0 |  | 1±2a | 0±0b | 0±0b |
|  |  | *Xanthomonadales* | *Xanthomonadaceae* | *Arenimonas* | 1.1±1 | 0.5±0 |  | **1.4±1** | 0.3±0 |  | 0.4±0a | 0.1±0ab | 0.1±0b |
|  |  |  |  | *Lysobacter* | 1±1 | 0.4±0 |  | **1.6±2** | 0.3±0 |  | 0.3±0 | 0.1±0 | 0.3±0 |
|  |  |  |  | *Pseudoxanthomonas* | 0.8±1 | 0.4±0 |  | **1.9±1** | 0.2±0 |  | 0.6±0b | 0.4±0b | 1.8±1a |
|  |  |  |  | *Rhodanobacter* | 0±0 | 0±0 |  | 0.2±0 | 0.2±0 |  | 0.3±0b | 0.6±1a | 0.1±0b |
|  |  |  |  | *Stenotrophomonas* | 0±0 | 0±0 |  | 0.1±0 | 0.2±0 |  | 0±0c | 0.3±0b | 3.1±2a |
|  | *Deltaproteobacteria* | *Myxococcales* |  |  | 2.3±1 | 1.2±1 |  | **3.4±1** | 1.1±0 |  | 3.5±1a | 1.1±0b | 1.2±1b |
| *Acidobacteria* | *Acidobacteria_Gp10* |  |  | *Gp10* | 2.9±1 | **6.4±1** |  | 2.3±0 | **8.5±3** |  | 2.2±1b | 6.6±1a | 3.6±1b |
|  | *Acidobacteria_Gp4* |  |  | *Gp4* | 7.9±1 | 8±2 |  | 5.2±0 | 4.7±1 |  | 8.1±3a | 6.1±1ab | 5.3±1b |
|  | *Acidobacteria_Gp6* |  |  | *Gp6* | 14.3±1 | **18.3±1** |  | 11.5±1 | **14.7±1** |  | 11±2a | 12.4±3a | 9±1b |
| *Firmicutes* | *Bacilli* | *Lactobacillales* |  |  | 0±0 | 0.1±0 |  | 0.1±0 | 0.4±0 |  | 0.1±0b | 0.4±0a | 0.6±0a |
|  | *Clostridia* | *Clostridiales* | *Clostridiaceae* | *Clostridium* | 0.4±0 | 0.5±0 |  | 1±1 | 1.2±0 |  | 1.3±0b | 1.7±1b | 4.3±1a |
|  |  |  | *Peptostreptococcaceae* | *Peptostreptococcus* | 0.2±0 | 0.4±0 |  | 0.4±0 | 0.4±0 |  | 0.4±0b | 0.5±0ab | 1.1±0a |
| *Bacteroidetes* | *Sphingobacteria* | *Sphingobacteriales* | *Cytophagaceae* | *Adhaeribacter* | 1.5±0 | 0.7±0 |  | 1.3±0 | 0.4±0 |  | 1.9±1a | 0.4±0b | 0.2±0b |
| *Actinobacteria* | *Actinobacteria* | *Actinomycetales* | *Microbacteriaceae* | *Leifsonia* | 0±0 | 0±0 |  | 0±0 | 0±0 |  | 0.1±0b | 0.1±0b | 0.5±0a |
| *Chloroflexi* |  |  |  |  | 2.3±1 | 3.1±0 |  | 5±1 | 5.9±0 |  | 4±1b | 4.8±1b | 8.5±2a |
| *Gemmatimonadetes* | *Gemmatimonadetes* | *Gemmatimonadales* | *Gemmatimonadaceae* | *Gemmatimonas* | 0.9±1 | 1.1±1 |  | 0.5±0 | **1.4±1** |  | 0.7±1c | 1.2±1b | 1.9±1a |
| *OP10* |  |  |  | *OP10_genera_*  *incertae_sedis* | 0.4±0 | 0.4±0 |  | 0.5±0 | 0.4±0 |  | 0.5±0b | 0.7±0ab | 1±0a |
| *Planctomycetes* | *Planctomycetacia* | *Planctomycetales* | *Planctomycetaceae* |  | 2.6±1 | 2.6±1 |  | 2.7±0 | 2.5±0 |  | 2.4±0ab | 2.6±0a | 1.8±0b |

Bold text indicates taxa with significantly higher relative abundance in SDZ-treated or untreated soils. a,b and c indicate taxa with significant difference in relative abundance between S0, S10 and S100 treated soils collected at day 193. Soils were collected 60 days after each manure application.


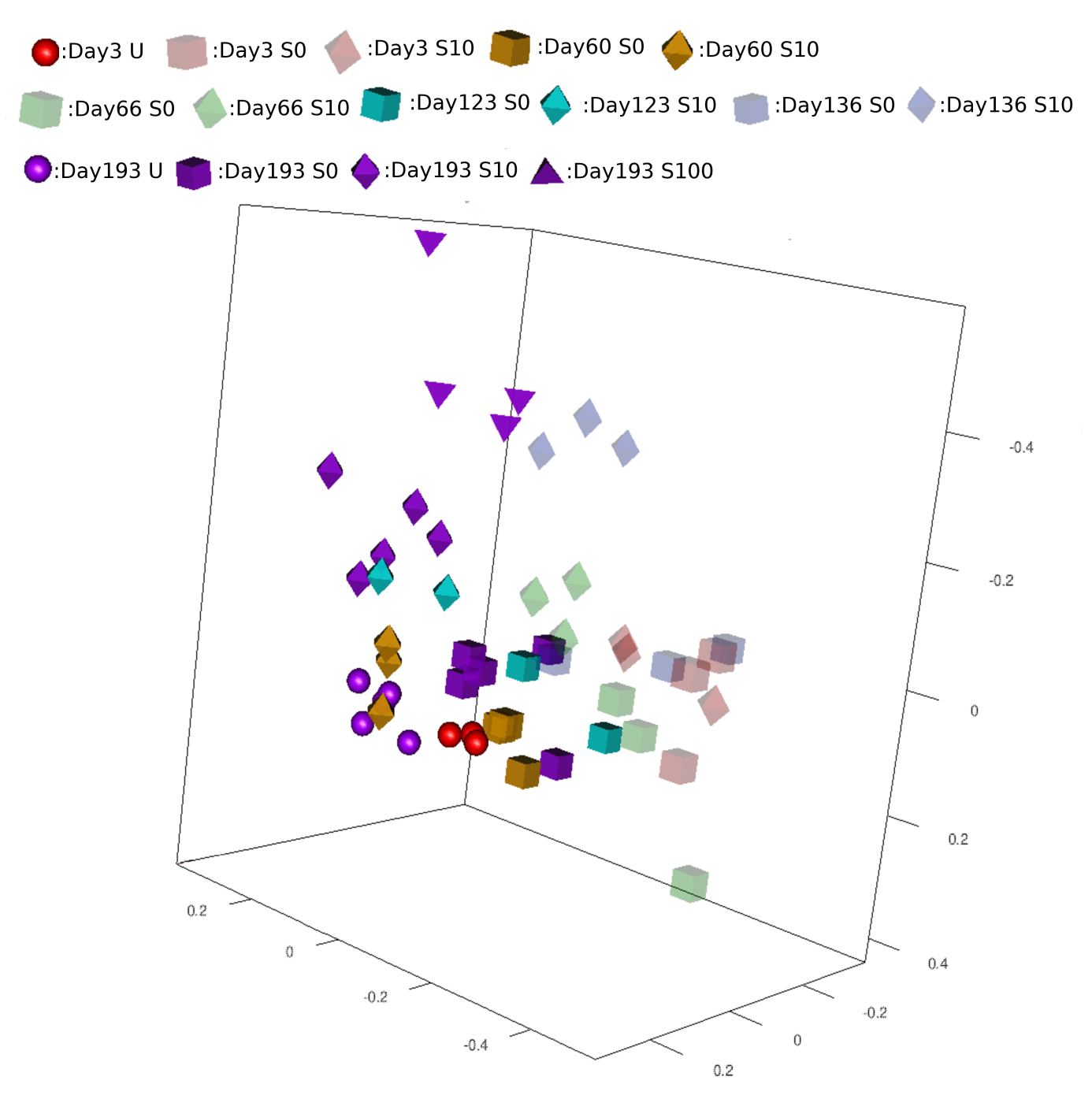
**Figure S1** Non metric multidimensional scaling based on OTU reports acquired by the forward primer sequencing. Balls: untreated soils; cubes: S0; octahedron: S10; pyramid: S100.
